# Addition of a carbohydrate-binding module enhances cellulase penetration into cellulose substrates

**DOI:** 10.1186/1754-6834-6-93

**Published:** 2013-07-03

**Authors:** Vimalier Reyes-Ortiz, Richard A Heins, Gang Cheng, Edward Y Kim, Briana C Vernon, Ryan B Elandt, Paul D Adams, Kenneth L Sale, Masood Z Hadi, Blake A Simmons, Michael S Kent, Danielle Tullman-Ercek

**Affiliations:** 1 Deconstruction Division, Joint BioEnergy Institute, Emeryville, CA 94608, US; 2 Department of Bioengineering, University of California, Berkeley, CA 94720, US; 3 Sandia National Laboratories, Livermore, CA 94550, US; 4 Sandia National Laboratories, Albuquerque, NM 87185, US; 5 Department of Chemical and Biomolecular Engineering, University of California, Berkeley, CA 94720, US; 6 Physical Biosciences Division, Lawrence Berkeley National Laboratory, Berkeley, CA 94720, US

**Keywords:** Cellulases, Endoglucanases, Carbohydrate-Binding modules, Cellulose model films, Neutron reflectometry

## Abstract

**Introduction:**

Cellulases are of great interest for application in biomass degradation, yet the molecular details of the mode of action of glycoside hydrolases during degradation of insoluble cellulose remain elusive. To further improve these enzymes for application at industrial conditions, it is critical to gain a better understanding of not only the details of the degradation process, but also the function of accessory modules.

**Method:**

We fused a carbohydrate-binding module (CBM) from family 2a to two thermophilic endoglucanases. We then applied neutron reflectometry to determine the mechanism of the resulting enhancements.

**Results:**

Catalytic activity of the chimeric enzymes was enhanced up to three fold on insoluble cellulose substrates as compared to wild type. Importantly, we demonstrate that the wild type enzymes affect primarily the surface properties of an amorphous cellulose film, while the chimeras containing a CBM alter the bulk properties of the amorphous film.

**Conclusion:**

Our findings suggest that the CBM improves the efficiency of these cellulases by enabling digestion within the bulk of the film.

## Background

The utilization of enzymes for the conversion of biomass into fermentable products has been demonstrated to be a viable and promising approach toward the development of cost-effective biofuels [1,2]. Cellulases catalyze the hydrolysis of β-1,4-glycosidic bonds in cellulose, the most abundant biomass polymer. Despite advances in protein expression and enzyme optimization, there is still a need for significant improvement with respect to cellulase exnzymatic activity at industrial process conditions, such as high temperature [3-7]. To that end, there have been some reported successes in engineering enzymes to be more active and stable at high temperatures [3,8,9], but there is still a need for additional improvement to further lower the costs of biofuel production [2,6,10]. Thermophilic cellulases, in particular, are promising due to their temperature stability but do not natively have the high activities required for efficient biomass hydrolysis [2,3,8].

A cellulase consists of a catalytic domain (CD) that often is linked to other modular accessory domains, including carbohydrate-binding modules (CBMs) [11], in many possible orientations and combinations [12-14]. CBMs are thought to 1) enhance the adsorption of CDs to their substrate [15]; 2) enable the alignment of cellulose fibrils for better docking of the CD [16,17]; and 3) modify substrate surfaces to facilitate enzymatic hydrolysis [18-20]. However, some molecular details are still unclear, particularly in relation to how the CBM affects enzyme-substrate interactions and activity on solid substrates.

Several studies have been carried out for the purpose of understanding cellulase-substrate interactions [21-28]. Ellipsometry and quartz crystal microbalance with dissipation (QCM-D) were used to correlate changes in mass and energy dissipation to structural changes of crystalline cellulose model films incubated with cellulases [23]. Josefsson et al. performed similar studies with QCM-D and atomic force microscopy which suggested that endoglucanases cause swelling of crystalline cellulose films [24]. Recently, analytical techniques have been applied to understand the interactions with amorphous films as well [27,28]. For example, Suchy et al. combined QCM-D, atomic force microscopy, and X-ray photoelectron spectroscopy to demonstrate that an endoglucanase and a cellobiohydrolase work uniformly within the entire volume of swollen amorphous cellulose films [27]. Neutron reflectometry (NR) has also been applied to differentiate between cellulases that are more active at the surface of an amorphous cellulose film and those that are active in the bulk of the same substrate [20,28]. We reasoned that these analytical techniques might be similarly applied to study the interactions of the CBM, CD, and cellulose substrate.

This study directly addresses the effect of addition of a CBM to a CD in terms of changes in enzyme activity on insoluble substrate and on alterations to both the surface and bulk properties of non-crystalline cellulose films. To examine these effects, we genetically fused a CBM from family 2a to two thermophilic endocellulases, Cel9A from *Alicyclobacillus acidocaldarius* and Cel5A from *Thermotoga maritima*, which do not naturally have a CBM. We observed that the addition of the CBM increases cellulase activity by up to three fold on insoluble cellulosic substrates. Data acquired with NR demonstrates that the wild type enzymes are active mainly on the surface of amorphous cellulose films, while their respective CBM-containing chimeras substantially alter the bulk properties of cellulose films. These findings suggest that addition of the CBM enhances the efficiency of the two CDs not only by enhancing adsorption to the surface of the film but also by enabling increased penetration into and digestion within the bulk of the film. We therefore propose a new model for CBM-enhanced insoluble cellulose degradation.

## Results and discussion

### Construction and characterization of chimeric cellulases

In this work, we set out to systematically investigate the effect of addition of a CBM to the CD of two thermophilic cellulases, Cel9A from *A*. *acidocaldarius* and Cel5A from *T*. *maritima*. Cel9A and Cel5A are endoglucanases that release cellobiose and cellotriose, respectively, as their primary hydrolysis product [29-31]. We fused these CDs to a family 2a CBM from the thermophilic exoglucanase E3 (UniProt Q60029) from *Thermomonospora fusca*. This CBM is natively at the N-terminus of a CD from glycosyl hydrolase family 6 [32,33] and is expected to bind crystalline cellulose [34]. The chimeras were constructed such that the CBM is at the C-terminus, with a 31 amino acid linker, denoted as “CD-CBM”. The CBM2a chimeras were also made with two additional linker lengths (12 and 47 amino acids) in order to examine the role of linker length on activity. Finally, mutants for which the catalytic activity is eliminated (knockouts) were made for both the wild type and chimeras with the 31 amino acid linkers (“CD_ko_-CBM” and “CD_ko_”). The enzymes, chimeras, and knockouts were overexpressed in *Escherichia coli* and purified to >80% purity.

Catalytic activity of the chimeric cellulases on carboxymethylcellulose (CMC) closely matched that of the corresponding wild type enzymes (Additional file 1: Figures S1 and S2). Optimal temperatures (T_opt_) and pHs were determined to be 75°C and pH 4.8 for Cel5A and its corresponding chimeras, and 65°C and pH 5.5 for Cel9A and its chimeras (Additional file 1: Figures S1 and S2). These values are similar to those reported previously for the wild type enzymes [35,36]. Polyacrylamide gel electrophoresis followed by Coomassie staining indicates no degradation of the chimera under standard assay conditions (Additional file 1: Figure S3).

### Enzymatic hydrolysis of insoluble substrates

**Figure 1:**
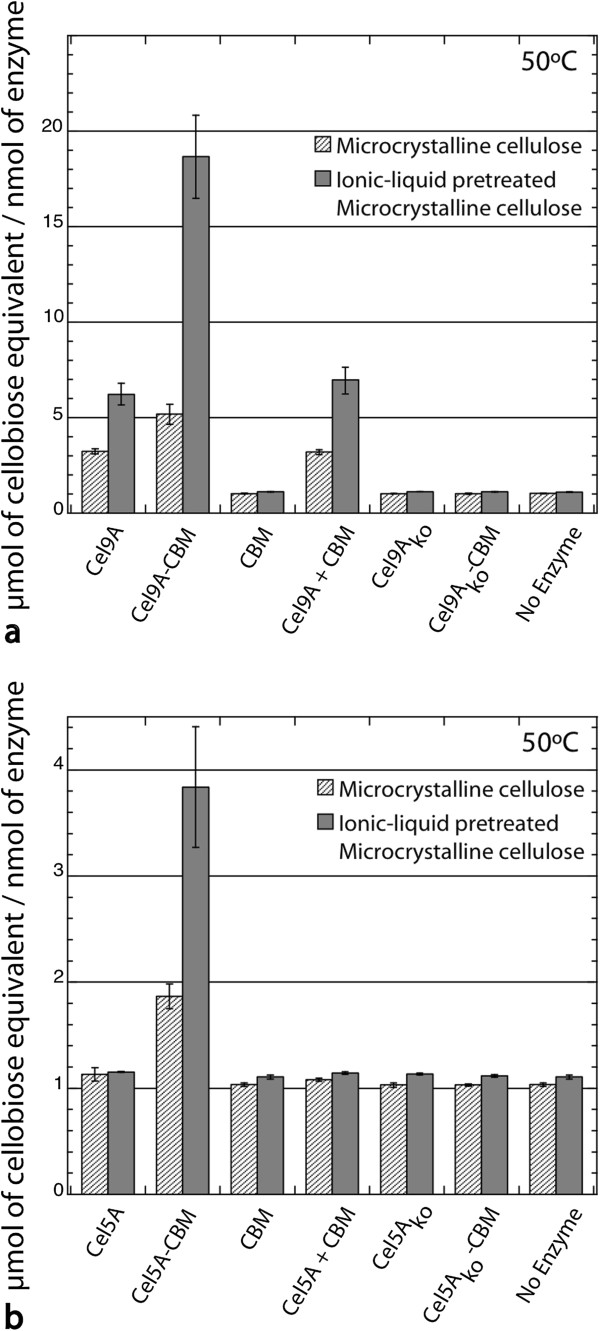
**Chimeric cellulases** (**CD**-**CBM**) **show higher soluble sugar production from insoluble microcrystalline cellulose (MCC).** Two insoluble substrates, microcrystalline cellulose and ionic liquid-pretreated microcrystalline cellulose, were used for this assay. **a)** Cel9A, Cel9A_ko_*, Cel9A-CBM, Cel9A_ko_-CBM*, CBM alone, and Cel9A + CBM untethered; **b)** Cel5A, Cel5A_ko_, Cel5A-CBM, Cel5A_ko_-CBM, CBM alone, and Cel5A + CBM untethered. Cellulases were loaded at 200 nM with either 50 mg/ml MCC or IL-MCC at 50°C for 24 h, and DNS assay was used to quantify sugar release with a cellobiose standard curve. *Inactive catalytic domain is indicated with the letters “ko”.

Enzymatic activities were measured *via* dinitrosalicylic acid (DNS) assay on microcrystalline cellulose (MCC, Avicel pH-101, Sigma) and ionic-liquid pretreated microcrystalline cellulose (IL-MCC), to represent model crystalline and non-crystalline cellulosic substrates. The chimeric cellulases gave rise to up to approximately three-fold increases in reducing ends produced from IL-MCC compared to their corresponding wild type CDs alone (Figure 1). Ionic liquid pretreatment is a promising method for biomass preparation that causes the structure of the cellulose to change from mostly crystalline to a mixture of cellulose II and amorphous cellulose [37], thereby enhancing downstream enzymatic hydrolysis [37,38]. Since CBMs have different binding preferences [13,14,39,40], we conclude that this CBM2a is more specific for cellulose sites accessible within the less crystalline substrate IL-MCC [37] than for sites within MCC. It should be noted that while the Cel9A CD liberated soluble cellodextrins from both MCC and IL-MCC, the Cel5A CD alone did not exhibit any significant activity above background on either substrate (Figure 1, Additional file 1: Figure S4). These results served as motivation to test for enzymatic hydrolysis of IL-MCC at higher enzyme loadings. At these higher enzyme loadings, the Cel5A CD did give rise to measurable cellodextrin release. Furthermore, the chimeric cellulases were again approximately three-fold more active than their respective CDs at every enzyme concentration on IL-MCC (Additional file 1: Figures S5, results reported in total production of cellodextrin). These experiments suggest that the CBM is the factor responsible for enhancing the cellulase activity of both chimeric constructs. This result is in agreement with the results of Kim et al., who also found up to three-fold increases in activity upon fusion of a CBM to certain CDs [9].

As a control, we also measured the enzymatic activities on these substrates using the same concentration of CBM with either Cel9A or Cel5A added as separate (non- fusion) proteins. For these controls we observed the same level of activity as for each respective CD alone (Figure 1); therefore, physical linkage to the CBM is required for enhanced hydrolysis. Hydrolysis assays were also performed with chimeric cellulases that had linker lengths of 12 and 47 amino acids. As there were no significant differences in activity between the 31 amino acid linker and the other two linkers for either chimera (p > 0.05 by two-tailed student’s t-test with equal variance for each; Additional file 1: Figure S4), the remaining experiments were carried out using the original 31 amino acid linker.

### Amorphous cellulose film characterization

To further explore the mechanism for the enhanced hydrolysis activity of the chimeras, we used neutron reflectivity to analyze changes in cellulose thin films upon incubation with the enzymes. The cellulose films were regenerated from trimethylsilyl cellulose (TMSC), and were largely amorphous as reported previously [20,41], permitting comparison to the model substrate IL-MCC used in the hydrolysis assays. The films swelled in aqueous buffer (with no enzyme added) by a factor of ~1.9 - 2.2. Two concentrations of TMSC were used in order to determine if the roughness of the film varied appreciably with film thickness, but no consistent trend was observed. The film thickness was 250 Å - 270 Å for 10 mg/ml TMSC, and 310 Å– 340 Å for 12 mg/ml TMSC. The density of the dried films varied from 1.25 to 1.35 g/cm^3^.

Other characteristics of these films have been reported previously [20]. NR data revealed that the films were highly smooth, and that swelling of the films was uniform except for some variation at the film-substrate interface.

### NR analysis of films incubated with cellulases

To aid interpretation of the NR results, we summarize here several effects expected upon interaction of endoglucanases with amorphous cellulose films.

i) Activity of endoglucanases at the surface of a film will release mass and result in a decrease in film thickness. ii) Activity of endoglucanases in the bulk of a film will result in an increase in water content. As endoglucanases digest they cleave β-1,4-glycosidic bonds randomly along cellulose chains. Enzymatic cleavage of bonds interior to cellulose chains will create free chain ends, whereas cleavage near the ends of cellulose chains will result in the release of soluble fragments. Since chain ends resulting from hydrolysis are hydrophilic by virtue of the hydroxyl group, both cases will result in increased water content. iii) Activity of endoglucanases in the bulk of a film may lead to an increase in film thickness. We assume that amorphous cellulose in an aqueous buffer will behave as a glassy polymer. For glassy polymers, the occupied volume will increase with an increase in the number of chain ends. This, and the increased hydrophilicity of chain ends, will both contribute to an increase in thickness upon the action of endoglucanases within the film. We note that hydrolysis occurring at chain ends, while increasing the water content, will not result in an increase in film thickness. iv). Adsorption of CDs and CBMs to cellulose will generally be weaker at higher temperatures due to entropic considerations.

We note that film expansion from activity within the bulk can be compensated by enzyme activity at the surface. Therefore, for enzymes that penetrate into the bulk of the film, the magnitude of the change (increase or decrease) in film thickness will be determined by i) the amount of bond scission interior to chains that creates chain ends relative to the amount of bond scission at chain ends that results in soluble fragments, and ii) the relative levels of activity within the bulk and at the surface of the film.

Finally, NR is highly sensitive to changes in the breadth of the film/solution interface. Broadening of that interface could occur due to enzymes digesting as they penetrate into the film, or could also result from film swelling. Lateral surface roughness and a composition gradient normal to the interface have identical effects on the reflectivity data [42,43].

**Figure 2:**
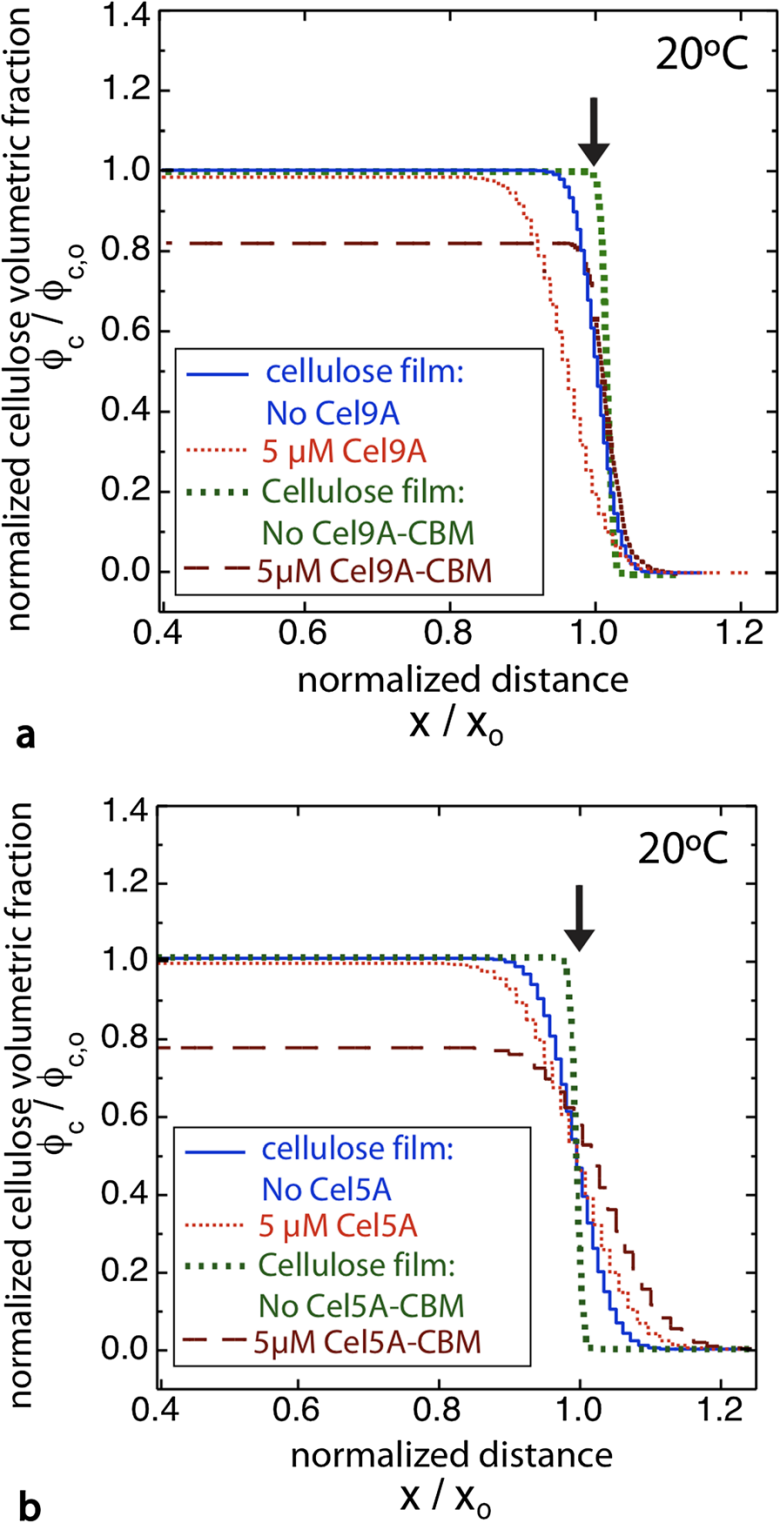
**Chimeric cellulases (CD-CBM) show greater penetration and bulk degradation of amorphous cellulose films at room temperature.** Cellulose volume profiles are shown for each cellulase. Amorphous cellulose films were incubated at room temperature with **a)** Cel9A or Cel9A-CBM, and **b)** Cel5A or Cel5A-CBM. Plots show the normalized cellulose volume fraction versus normalized distance from the silicon oxide surface. Due to variance in film preparation the baseline (film prior to enzyme addition) for every cellulose film assay is shown. Arrows indicate the cellulose-buffer interface. Brown dotted line shows the extrapolation of the cellulose profile for Cel9A-CBM after the enzyme layer at the surface of the film was removed.

With these considerations in mind we now discuss the NR results for the CDs and chimeras. In each case, initial (prior to adding enzymes) and final volume fraction profiles are compared. Measurements were taken until the activity had slowed such that little or no change in the NR data was observed on the time scale of several hours; the profile for each experiment is the final profile, generated from the last NR measurement. For Cel9A and Cel5A incubated against cellulose films at room temperature (RT), the cellulose volume fraction profiles (Figure 2, NR data shown in Additional file 1: Figures S6-S7) show relatively small changes. Cel9A and Cel5A removed only 5% and 1% of the cellulose, respectively (Table 1). The volume fraction profiles indicate that cellulose was removed primarily from the surface of the films. In contrast, far more substantial changes were observed in films incubated with the chimeras. First, much greater mass loss resulted (12% and 16% for Cel9A-CBM and Cel5A-CBM, respectively). Second, much greater digestion occurred within the bulk of the films, as reflected by large increases in water content. Third, for Cel5A-CBM the cellulose-buffer interface moved to greater depths, indicating film expansion at RT. This expansion, despite the substantial loss in cellulose mass, is qualitatively different from the changes observed for the Cel5A CD alone. Taken together, these results demonstrate that the chimeric enzymes penetrate into, and are active within, the cellulose films to a far greater extent than the CDs alone. We emphasize that the final profiles were measured after activity had slowed dramatically, and so the lack of activity of the CDs within the bulk of the film is a true limitation of the enzyme and is not due to a slower rate or insufficient time. Moreover, we repeated the experiment with five-fold less Cel9A-CBM (1 μM) and the final profile was nearly the same as at 5 μM (data not shown).

**Table 1:** Observations of cellulose films exposed to different cellulases

**Cellulase**	**Percentage of mass/area remove**		**Film swelling**
	**RT**	**Topt**	
Cel9A	5	11	No
Cel9A-CBM	12	32	Yes
Cel5A	1	4	No
Cel5A-CBM	16	35	Yes

Chimeric cellulases show higher cellulose removal and swelling of the amorphous cellulose film. The table above summarizes the neutron reflectivity (NR) results of the amorphous cellulose films exposed to several wild type and chimeric cellulases at room temperature. The mass/area removed is determined by integrating the cellulose volume profiles. Film swelling is the increase of film thickness above baseline observed after the film is exposed to the cellulase. RT and Topt stand for “room temperature” and “optimal temperature”, respectively. All standard deviations are within 10%.

**Figure 3:**
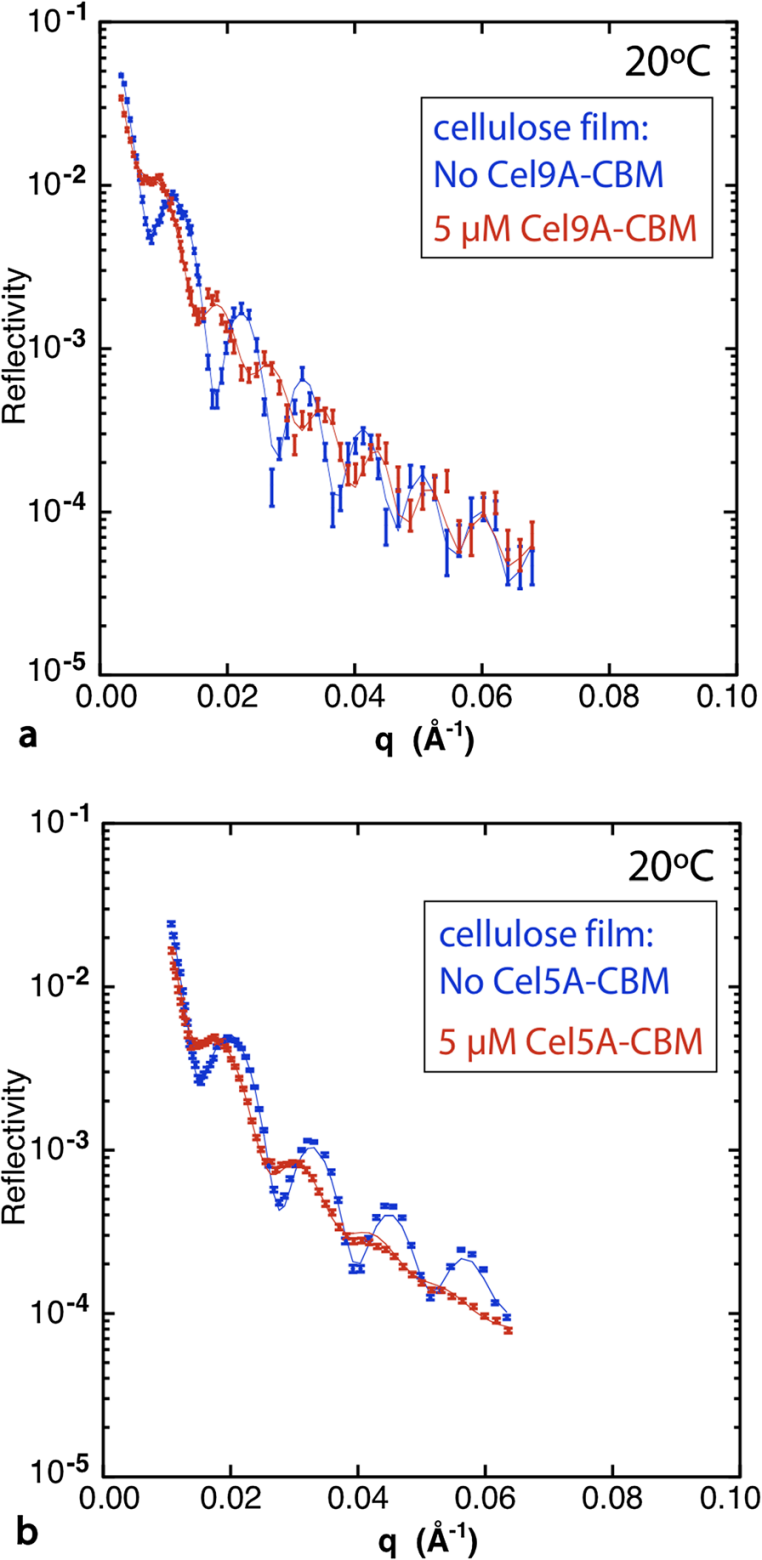
**Figure 3 Neutron reflectivity data from cellulose films exposed to the chimeric cellulases Cel9A-CBM (a) and Cel5A-CBM (b) at 5 μM and 20°C.** The fringe pattern, related to the thicknesses of the various layers, is distinctly different for the two chimeras: the fringes decay uniformly with qz for Cel5A-CBM, whereas for Cel9A-CBM the first three fringes are damped relative to the fringes at higher qz. The latter pattern indicates a distinct layer of enzyme adsorbed at the surface of the film.

For Cel9A-CBM a fringe pattern that is distinctly different from that for Cel5A-CBM resulted, that indicates a layer of protein adsorbed to the film surface (Figure 3 and Additional file 1: Figure S8). The additional layer at the film surface is clearly identified as a layer of protein because the scattering length density (SLD) value for that layer is much lower than that of the swollen cellulose film. This additional layer is not observed for the Cel9A CD alone or for the Cel5A-CBM chimera. Therefore this layer is likely due to strong CBM binding at the surface along with an inherent property of the Cel9A CD that inhibits its penetration (*e*.*g*. larger size that slows its entry into the bulk of the film). Thus for Cel9A-CBM the results indicate substantial penetration into the bulk as well as a distinct layer of enzyme remaining at the surface. Film expansion was not observed for Cel9A-CBM as for Cel5A-CBM, but it is possible that for Cel9A-CBM film expansion was compensated by activity at the surface of the film due to the adsorbed layer of enzymes. The cellulose profile shown in Figure 2 for Cel9A-CBM was obtained by truncating the full SLD profile (Additional file 1: Figure S8a) at the interface between the cellulose film and the layer of adsorbed enzyme.

**Figure 4:**
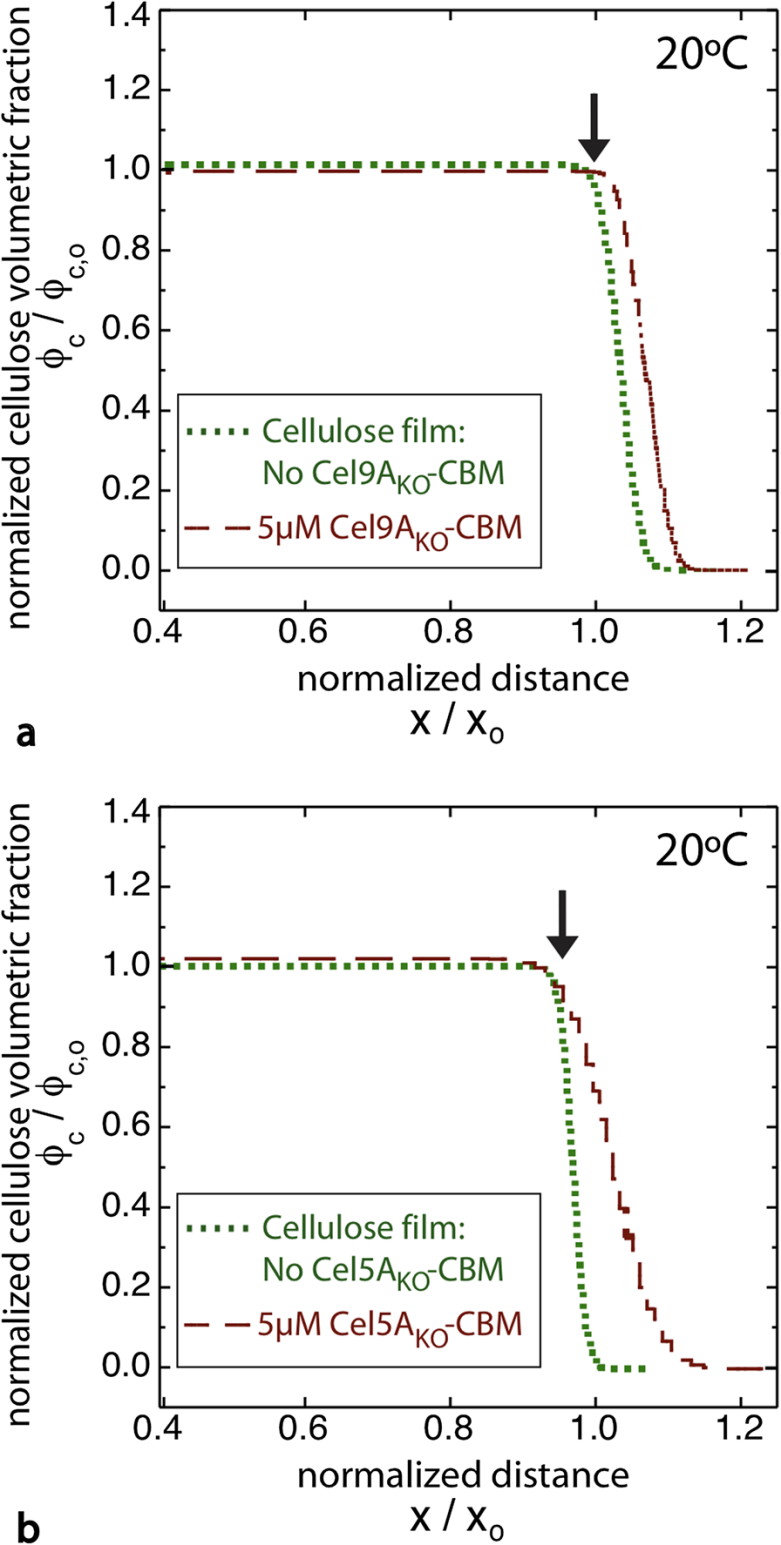
**Cellulose volume profiles are shown for chimeric cellulases with inactive catalytic domains (CD**_**KO**_**-CBM).** Amorphous cellulose films were incubated at room temperature with **a)** Cel9A_KO_-CBM, and **b)** Cel5A_KO_-CBM. Plots show the normalized cellulose volume fraction versus normalized distance from the silicon oxide surface. The profile for the film prior to enzyme addition is also shown. Arrows indicate the cellulose-buffer interface. Brown dotted line shows the extrapolation of the cellulose profile for Cel9A_KO_-CBM after the enzyme layer at the surface of the film was removed.

As controls, we also examined the effect of incubating the film with the knockout chimeras, which are inactive due to mutations in the active site residues (Figure 4, Additional file 1: Figures S9 and S10). With each knockout chimera, the film expands somewhat (~30 Å for Cel5A_ko_-CBM and ~15 Å for Cel9A_ko_-CBM), supporting the conclusion that the protein is penetrating into the bulk of the film. In addition we observe again that a layer of protein adsorbs to the film surface for Cel9A_ko_-CBM, but not for Cel5A_ko_-CBM (Additional file 1: Figures S9b, S10b). There is negligible change in the cellulose volume fraction and water content of the films, as expected for inactive enzymes. While some water displacement is expected from protein entering the film, this will be offset by the increased hydrophilicity of the protein relative to cellulose. We also incubated a film with the same concentration of CBM alone at RT and at 65°C (Additional file 1: Figures S11, S12), and incubated another film with a mixture of Cel9A and CBM (Additional file 1: Figure S13). For CBM alone a small but definite film expansion (~10 Å) was observed at each temperature. Again, this is consistent with CBM localization within the bulk of the film, and slight swelling due to the hydrophilicity of the protein. For the mixture of Cel9A and CBM the effects were far weaker than for the chimera. The water content in the bulk of the film was only slightly greater than for the CBM alone, and the increase in film thickness was comparable to that for the CBM alone (Figure 3). These results suggest that localization of the CBM within the bulk film may slightly increase the penetration of Cel9A, but not nearly to the level as for the chimera.

**Figure 5:**
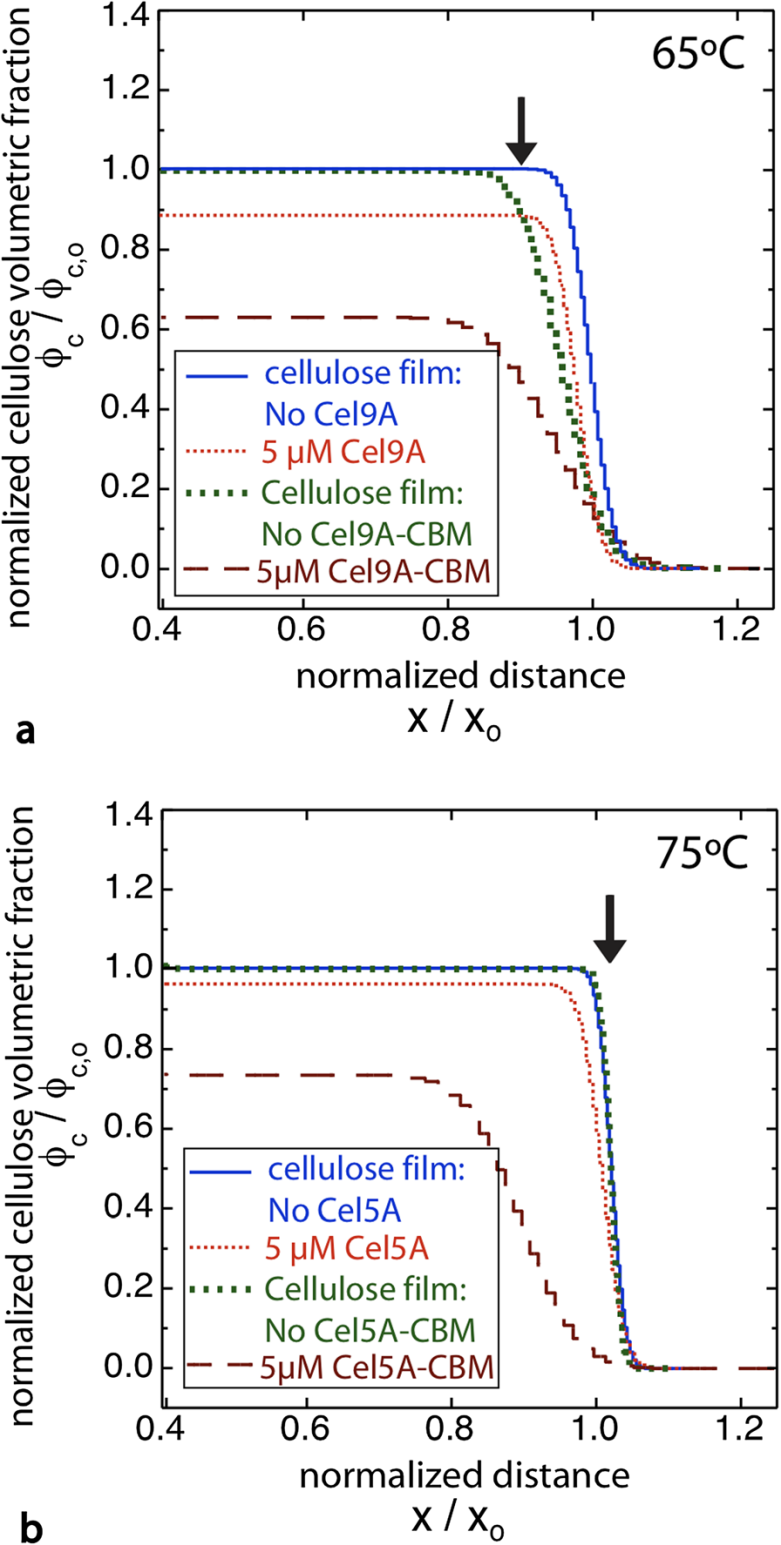
**Chimeric cellulases** (**CD**-**CBM**) **show greater penetration and bulk degradation of amorphous cellulose films at T**_**opt**_**.** Cellulose volume fraction profiles at T_opt_ (65°C for Cel9A and its chimera, and 75°C for Cel5A and its chimera) are shown for each cellulase. Amorphous cellulose films were incubated with **a)** Cel9A or Cel9A-CBM, and **b)** Cel5A or Cel5A-CBM. The profile for the film prior to enzyme addition is also shown. Arrows indicate the cellulose-buffer interface.

**Figure 6:**
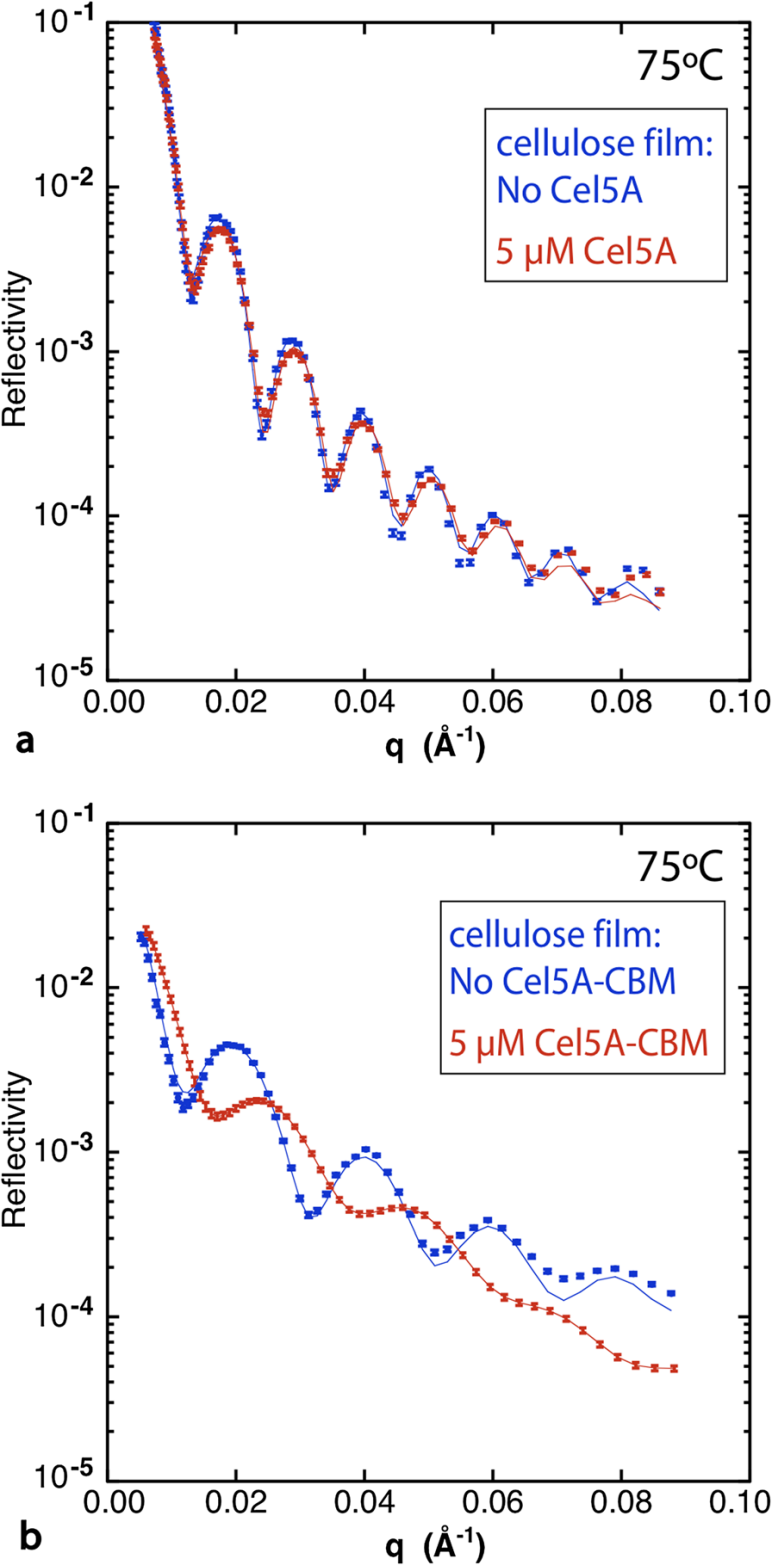
**Neutron reflectivity data from cellulose films before and after incubation at 75**°**C with a) 5 μM Cel5A and b) 5 μM Cel5A-CBM.**

At T_opt_ (65°C for Cel9A and 75°C for Cel5A) the results again show much greater activity within the bulk of the film for the chimeras relative to the wild-type enzymes as indicated by greatly increased water content (Figure 5). In addition, a large increase in the interfacial gradient occurred with the chimeras, whereas little change in the interfacial gradient resulted with the CDs. The increase in breadth of the film-solution interface is very obvious in the NR data as the fringes are severely damped at higher q_z_ values (Figure 6, Additional file 1: Figure S14). The large interfacial gradient in each case is consistent with the hydrolysis of bonds as the chimeras penetrate into the film as well as film swelling.

At T_opt_ the change in film thickness between Cel5ACBM and Cel5A is distinctly different than at RT. We suggest that this may be due to the competing effects of bulk and surface activity. We note that there is a process and rate constant associated with each domain of the chimeras, and the relative rates of CD activity (R _CD_) and CBM-driven penetration of the chimera into the film (R _pen_) will affect the resulting film structure. When R _CD_ / R _pen_ < < 1 the chimeras penetrate and equilibrate throughout the film before hydrolytic bond cleavage occurs. In that case bond cleavage occurs uniformly throughout the film, and swelling resulting from bond scission within the bulk will be maximal. The data for Cel5A-CBM at RT appears to correspond to this case. When R _CD_ / R _pen_ > 1 the chimeras hydrolyze bonds as they penetrate into the bulk, beginning at the surface. The result for Cel5A-CBM at T_opt_ seems to correspond to this case in that at T_opt_ the film thickness for Cel5A-CBM is substantially reduced relative to the thickness of the as-prepared film. A similar trend with temperature was reported previously in NR data of Cel45A from *Humicola insolens*, which contains a CBM, digesting amorphous cellulose films [28]. For Cel9A-CBM at T_opt_ a distinct layer of enzyme is not detectable at the surface as is the case at RT. Greater penetration by Cel9A-CBM at the higher temperature could be explained by greater mobility of the cellulose chains. While there is greatly increased water content in the bulk of the film indicating penetration and digestion by Cel9A-CBM, the film thickness only decreased slightly from the as-prepared film and there is a very large gradient at the film-solution interface. In this case digestion in the bulk of the film by Cel9A-CBM appears to have led to sufficient film expansion to compensate for thickness loss due to surface activity. A particularly large film expansion is also consistent with the large gradient at the film-solution interface.

Finally, since the binding domain is the same for the two chimeras, comparing the results reveals differences due to the two CDs. The first striking difference is the presence of a dense adsorbed protein layer at the surface for films incubated with Cel9A-CBM or the Cel9A-CBM_KO_ at RT, but not for those incubated with Cel5A-CBM or Cel5A_KO_-CBM. We suggest that this is due to the greater size of Cel9A (MW = 62 kDa) compared with that of Cel5A (MW = 40 kDa), which impedes Cel9A from entering the film. While size is the attribute that we expect to contribute the most, other differences between the two enzymes, such as the presence of the Ig-like domain or differences in the nature of the surface-exposed residues, could cause the formation of this adsorbed protein layer. The second difference is the reduced film thickness for Cel5A-CBM at T_opt_ along with the greater interfacial roughness for Cel9A-CBM. One possible explanation consistent with these data is that the activity of Cel9A results in greater swelling in the bulk of the film than does Cel5A. The third difference is that the proportional increase in mass released from the amorphous films for chimera compared with CD was greater for Cel5A than for Cel9A. At T_opt_ for Cel9A and Cel5A the cellulose mass decreased by 11% and 4%, respectively, while for Cel9A-CBM and Cel5A-CBM the cellulose mass decreased by 32% and 35%, respectively (Table 1). So while three times as much mass was released by Cel9A-CBM as by Cel9A, roughly nine times as much mass was released by Cel5A-CBM as by Cel5A. This difference is due to the very low amount of mass released by Cel5A, while the chimeras released about the same amount of mass. The lower mass loss with Cel5A is likely due to a lower adsorption affinity for Cel5A than for Cel9A.

While the fold increase in cellulose mass released for Cel9A-CBM / Cel9A from NR is in good agreement with that obtained from the bulk saccharification assay on the IL-MCC substrate, the fold increase in cellulose mass released for Cel5A-CBM / Cel5A from NR is much greater than that released in the bulk saccharification assay on IL-MCC. This could be due to differences in the two assays. We note that IL-MCC is a mixture of amorphous and crystalline cellulose (cellulose II) [37], whereas the NR study involved films of amorphous cellulose with no detectable cellulose II. Another important difference in the assays is the fact that the bulk saccharification assays involved shaking/mixing whereas the NR study was performed in the absence of mixing.

### Proposed mechanism of CBM enhancement

Reese *et al*. first put forth a mechanism for cellulose degradation that involved a domain for cleavage or disruption of nonglycosidic linkages, in addition to the hydrolytic domain that cleaves the beta-1,4-glucosidic bond [44]. The non-hydrolytic domain could be CBMs, expansins, swollenins, or some other factor (for an extensive review, see [45]). For crystalline cellulose, the work of many research groups have led to a proposed mechanism in which the CBM is a swelling factor that helps to separate the glucan chains from the crystalline surface *via* the disruption of hydrogen bonds, resulting in layer-by-layer degradation [19,46]. Nonetheless, CBMs that are specific for non-crystalline cellulose also have been shown to increase cellulase activity on amorphous cellulose (Figure 1, and references [9,47]). The mechanism for this enhancement is largely unexplored, but is primarily attributed to the higher binding affinities that the CBMs confer to the enzymes [16,47-49].

**Figure 7:**
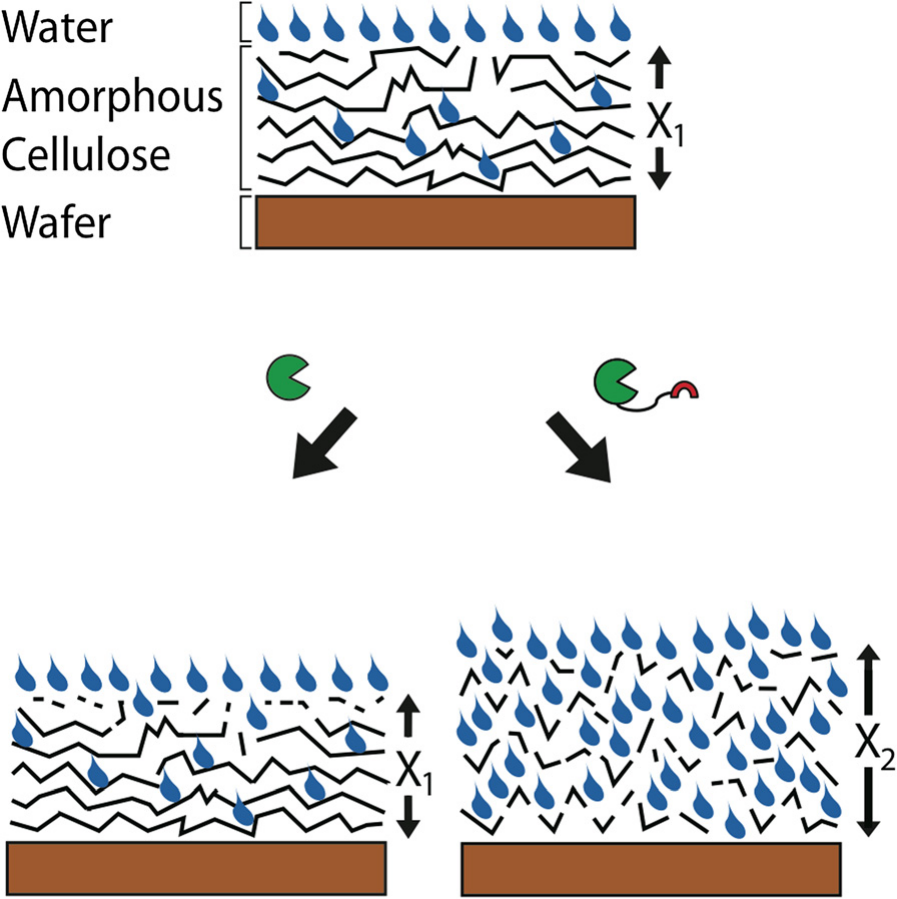
**Proposed model for chimeric cellulase degradation of amorphous cellulose substrates.** Wild-type cellulases (without CBM) are limited to surface degradation, while physical linkage to CBM permits penetration into the bulk of the substrate. Hydrolysis within the bulk creates sites for water coupling and enhanced enzymatic access to additional substrate, resulting in swelling.

In the present study the wild type enzymes are primarily active at the amorphous cellulose film surface. However, for the chimeric fusions containing a CD and the CBM from *T*. *fusca* E3 we observe a swelling of the cellulose film and substantial changes to its bulk properties upon incubation. These results point to a mechanism of amorphous cellulose disruption by the CBM akin to the cellulose “swelling factor” proposed in the model by Reese *et al*. [44] Taken together, the cellulose hydrolysis and NR data indicate that the addition of the CBM increases binding to cellulose and facilitates penetration into the bulk of the solid cellulose substrate (Figure 7). This enzyme penetration is driven by the interaction of the CBM with the cellulose chains. The CBM may disrupt hydrogen bonding between cellulose chains, or the increased affinity for cellulose provided by the CBM may overcome the energetic barrier to deform the cellulose chains as needed to allow the CD to penetrate into the film. As the enhanced activity requires physical tethering of the two domains, we suggest that the binding affinity of the CBM also still plays a role, providing the fused CD substantial time to access the newly available sites in the bulk of the cellulose substrate.

## Conclusions

The fusion of a CBM to the wild type cellulases Cel9A and Cel5A enhanced their activity as much as three fold on the insoluble lignocellulosic substrates MCC and IL-MCC. This activity enhancement can be explained as a change in mechanism in which the CBM increases binding to cellulose and dramatically enhances the penetration of the cellulases into the bulk of the cellulose. We and others [9] also observe that CBM addition has varying synergistic effects depending on the CD chosen. Additional work is needed to determine the parameters required for a CBM to enhance access to the bulk cellulose, and to explore the effect of CD choice and orientation on our proposed mechanism.

## Methods

### Strains and growth conditions

All cloning was carried out in DH10B and expression in BL21 (DE3) Star cells (Invitrogen). Cells were grown at 37°C in 2X YT media with 100 μg/ml kanamycin or 100 μg/ml carbenicillin as appropriate, unless otherwise noted.

### Cellulase genes and vector construction

Cel9A from *Alicyclobacillus acidocaldarius* (Cel9A) was initially obtained as a gift from K. Eckert (Humboldt-Universität zu Berlin, Institut für Biologie/Bakterienphysiologie, Germany) [29,31]. Its open reading frame was amplified and cloned into the pENTR221 vector (Invitrogen) modified to contain a thrombin cleavage site and ten consecutive histidine residues on its N terminus (N-terminal 10X His-tag) for affinity purification with nickel columns. The creation of the *cel5A* gene from *Thermotoga maritima* (encoding Cel5A) was described previously [30]. Amplification and the cloning process of the CD was analogous to that of the Cel9A CD.

The Cel9A- and Cel5A-CBM chimeras were created by combining three distinct sequences: genes encoding the CD, a synthetically designed linker region, and the CBM. The CBM (UniProt Q60029), found in an exoglucanase from *Thermotoga fusca*, was synthesized by GenScript Corporation. The linker was designed to be rich in proline and threonine, and is based on the sequence of a linker in endoglucanase N in *Erwinia carotovora*. Only the ends of this sequence were modified for overlapping polymerase chain reaction (PCR) and efficient cloning. The DNA encoding the linker was created using PCR assembly of multiple oligonucleotides (IDT, integrated Technologies). (Oligonucleotide sequences: forward (5’-GCTAACCTGGGCGGTGGTGATACTCCGACTACCCCTACCACCCCGACCGAACCGACTAAC-3’) and reverse (5’-ACCACCGGTGGCCGGCGTGGTGCCGTTGCCCGGGTTAGTCGGTTCGGTCGGG-3’). All three parts (Cel9A/Cel5A, CBM and linker) were amplified and then assembled by single overlap extension PCR. The resulting product was ligated into the modified pENTR221 vector as described above.

Chimeras also were constructed with 12 amino acid and 47 amino acid linkers, based on the sequence of the linker in CelE of *Caldicellulosiruptor sp*. Tok7B.1 and the endoglucanase/exoglucanase B from *Caldocellum saccharolyticum*, respectively. The linkers were altered slightly to include identical sequences at either end (5’-GCTAACCTGGGCGGTGGT-linker-CGGCCACCGGTGGT-’3) for efficient cloning via overlap PCR. The small linker (12 amino acids) was assembled by annealing of the two primers: forward (5’-GCTAACCTGGGCGGTGGTACGCCGGCCACCGGTGGT-3’) and reverse (5’-ACCACCGGTGGCCGGCGTACCACCGCCCAGGTTAGC-3’) in a decreasing temperature step gradient process from 98°C to 4°C. No polymerase or dNTPs were added to the mix. The large linker (47 amino acids) was assembled *via* overlap PCR using the following four primers:

forward-1 (5’-GCTAACCTGGGCGGTGGTCCGACCCCAACCCCAACTCC-3’)

reverse-1 (5’-GTCGGCGTCGGAGTCGGGGTCGGGGTAACCGTGACCGTTGGAGTTGGGGTTGGGGTC-3’)

forward-2 (5’-CGACTCCGACGCCGACGCCAACCGTTACCGCAACCCCGACTCCAACCCCTACTCCGGTG-3’)

reverse-2 (5’-ACCACCGGTGGCCGGCGTAGACACCGGAGTAGGGGTTGG-3’)

The fully assembled CD-linker-CBM chimeras were then constructed as described above.

Catalytic knockout versions of each enzyme were also constructed using site directed mutagenesis to introduce the following changes: E515Q in Cel9A and E136Q in Cel5A; the corresponding amino acids were also changed in Cel9A-CBM and Cel5A-CBM chimeras. Site-directed mutagenesis was achieved by carrying out PCR of the entire plasmid using the following primers: Cel9A forward primer (5’-GGACAGCTACTCGACCAACCAAGTCGCCGTCTACTGGAATTC-3’); Cel9A reverse primer (5’-GAATTCCAGTAGACGGCGACTTGGTTGGTCGAGTAGCTGTCC-3’); Cel5A forward primer (5’-GTTTTTCGAAATTCTGAACCAGCCGCATGGGAACCTGAC-3’); and Cel5Areverse primer (5’-GTCAGGTTCCCATGCGGCTGGTTCAGAATTTCGAAAAAC-3’).

All constructs were later passed into pDEST42 following standard Gateway cloning protocols (Invitrogen). The final constructs were verified by sequencing.

### Enzyme expression and purification

Cellulases were expressed in BL21 (DE3) Star cells in 2X YT media supplemented with 100 μg/ml of carbenicillin. Cells were grown at 37°C until reaching an OD600 of 0.7 at which time 0.5 mM IPTG was added to induce expression, and they were incubated with shaking at 18°C for 24 h. Cells were then pelleted down at 12,000 × g for 10 min at 4°C. The recombinant proteins were extracted from cell pellets using BugBuster (Novagen) according to the protocol with the following additions: 25 U benzonase/mL BugBuster (Novagen), 1 U r-lysozyme/mL BugBuster (Sigma), and 1× protein inhibitor cocktail V (EDTA-free) (Calbiochem). Cell debris was pelleted by centrifugation at 16,000 × g for 15 min at 4°C. The lysate was then incubated in a 50°C water bath for 15 min. Any denatured proteins were pelleted by centrifugation at 16,000 × g for 15 min at 4°C. The cellulases were affinity purified from the soluble fraction using gravity nickel columns (His GraviTrap, GE Healthcare) according to the manufacturer recommendations. Briefly, the columns were pre-equilibrated with washing buffer (20 mM sodium phosphate, 1 M NaCl, 20 mM imidazole, pH 7.4). The supernatant of the lysate described above was run twice through the equilibrated columns. Columns were washed with 10 ml of washing buffer. Enzymes were eluted by increasing the imidazole concentration to 500 mM. The eluted cellulases were buffer-exchanged using gravity desalting columns (PD-10 Desalting Columns, GE Healthcare) into a buffer consisting of 20 mM Tris, 150 mM NaCl, pH 7.4. Purity of the cellulases was determined by polyacrylamide gel electrophoresis. Concentration of the enzymes was determined by bicinchonic acid colorimetric assay (Pierce Thermo Scientific). The final protein concentrations were at least 1.3 mg/mL for each protein. The polydispersity of the purified cellulases was measured by dynamic light scattering on a DynaPro Plate Reader (Wyatt Technologies).

### Ionic liquid pretreatment

Ionic liquid pretreatment was carried out as previously described [50]. Briefly, the microcrystalline cellulose was treated with 1-ethyl-3-methylimidazolium acetate (Sigma-Aldrich, St Louis, MO, USA) at a loading of 3% (w/v) cellulose and heated at 160°C for three hours. The pretreated material was washed with deionized hot water (80°C). Samples were centrifuged at 10,000 × g for 20–25 min and were washed in this way five times. This has been shown to be sufficient for removal of ionic liquids [50]. Samples were freeze-dried in a lyophilizer (Labconco 12L Freeze Dryer).

### Enzymatic hydrolysis assays

Enzymatic hydrolysis assays were carried out as described before [35] with slight changes to the protocol. To determine the optimal temperatures and pHs of the cellulases, enzyme was added at 25 nM to a solution of 50mM citric buffer with 1% CMC in 96 well PCR plates to a total volume of 200 μl. The solution pH was varied incrementally from 4–8 (Additional file 1: Figures S1-S2) and the samples were incubated in a thermocycler for 30 minutes at temperatures varying from 10-95°C. After incubation, the soluble sugar content of all samples was quantified by colorimetric DNS assay (see below) [51].

For enzymatic hydrolysis assays on insoluble substrates, enzyme was added at 200 nM to citric buffer containing 20 mg/ml substrate to a total volume of 500 μl in micro-tubes with caps. Cellulases were tested at the optimal pH: 4.8 for Cel5Aand 5.5 for Cel9A. All samples were incubated for 24 hrs at 50°C and 1400 rpm in a bench-top thermomixer (Eppendorf AG 22331 Hamburg). There are some reports of shear stress inactivation, particularly of exoglucanases [52-56]. We did not observe any significant inactivation at this speed compared to lower speeds for the endocellulases in this study. After incubation, all samples were spun down and the supernatant collected to determine the concentration of soluble sugars using the DNS assay described below.

### Quantification of reducing sugar concentrations

The concentration of reducing ends in the samples was determined by DNS assay as previously described [51]. Briefly, 60 μl of 2× DNS reagent (1 g DNS, 30 g KNa tartrate, 20ml 2 N NaOH in 100 ml total volume) were mixed with 60 μl of the samples supernatant. Samples were well mixed and incubated at 95°C for five minutes. Samples were allowed to cool down to room temperature and their absorbance read at a wavelength of 540 nm (Molecular Devices, SpectraMax M2). Concentrations were calculated by comparison of absorbance to a cellobiose standard curve.

### Preparation of cellulose films

The preparation of uniform regenerated cellulose films sufficiently smooth for NR measurements has been previously reported [20]. In summary, regenerated cellulose films for NR were prepared by spincoating and conversion of precursor films of TMSC on polished silicon wafers (diameter = 75 mm, thickness = 5 mm). Preparation of TMSC has been described previously [57,58]. The wafers were cleaned in a solution of sulfuric acid/ 30% by volume hydrogen peroxide, 7:3 by volume (Piranha solution), followed by UV/ozone treatment for 20 min.

TMSC was spin-coated onto the cleaned silicon substrates with a spinning speed of 4000 rpm from solutions of 10 mg/ml or 12 mg/ml in toluene. The TMSC films were converted into cellulose by exposing them to vapors of 0.5 N HCl solution for 15 minutes in an enclosed container. This practice resulted in complete conversion and ultrasmooth films, as reported previously [20]. Film thicknesses ranged from 240 to 340 Å for the different solution concentrations as determined from X-ray reflectivity and NR and increased by a factor of 1.9 – 2.2 upon incubation with an aqueous buffer solution. The films were amorphous, as no diffraction peak was detected by grazing incidence X-ray diffraction [20].

### Neutron reflectivity

NR studies were performed on the SPEAR reflectometer (Lujan Center/LANSCE), Liquids (SNS/ORNL), and NG1 (NIST) reflectometers. The SPEAR and Liquids reflectometers operate in the time-of-flight mode where a band of wavelengths impinge onto the film-buffer interface by passing through the silicon wafer and are resolved at the detector based on their time-of-flight. Data collected from several incident angles were merged together. The measurements on NG1 were performed using a wavelength of 0.475 nm and varying angles of incidence. The data are plotted as the ratio of reflected to incident intensity as a function of momentum transfer q_z_ = (4π/λ) sinθ, where θ is the angle of incidence with respect to the plane of the film and λ is the wavelength [42]. The precise form of this curve is determined by the profile of the in-plane averaged scattering length density (SLD) normal to the surface. The SLD is determined by the atomic composition and the density [43]. The spacing between minima or maxima (fringes) on the q_z_ scale is related to the film thickness. The magnitude of the fringes is related to the volume fraction of water and cellulose within the film. Progressive dampening of fringes with increasing q_z_ indicates roughening or broadening of the solution film interface. The NR data were analyzed using the Ga_refl program based on the optical matrix method. Ga_refl is available at http://www.ncnr.nist.gov. Analyses were performed with free-form models involving a small number of slabs. Fitting reflectivity data results in defining a family of SLD curves that are consistent with the data. The uncertainty in the fitted profiles was determined by a Monte Carlo resampling procedure in which a large number (1000) of statistically independent sets of reflectivity data were created from the original data set and the error bars from the counting statistics. The result is a range of values for each fit parameter that is consistent with the statistics of the original data. This method has been reported in detail elsewhere [59]. The SLD profile bands for Cel5A and Cel5A-CBM at RT are given in the supporting information as a representative example (Additional file 1: Figure S15). The cellulose volume fractions (Figures 2, 4 and 5, Additional file 1: Figures S11b, S12b, S13b) were determined from the SLDs of the buffer solution, the swollen cellulose film, and pure cellulose using the following relation:

(1) $$\begin{array}{ll} {\left(\mathrm{SLD}\right)}_{\mathrm{meas}}= & \phi_{\mathrm{cellulose}}{\left(\mathrm{SLD}\right)}_{\mathrm{cellulose}}\\ & +\left(1-\phi_{\mathrm{cellulose}}\right){\left(\mathrm{SLD}\right)}_{\mathrm{buffer}} \end{array}$$

where (SLD)_meas_ is the measured SLD for the swollen film, ϕ_cellulose_ is the volume fraction of cellulose, (SLD)_cellulose_ = 1.67 × 10^-6^ Å^-2^ is the SLD of pure cellulose (C_6_H_10_O_5_) [20], and (SLD)_buffer_ = −0.54 × 10^-6^ Å^-2^ is the SLD for aqueous buffer. In using this equation, additivity of volumes is assumed. The amount of cellulose per unit area within each film was obtained by integrating the cellulose volume fraction profiles.

Before each measurement, the regenerated cellulose film was allowed to equilibrate with sodium acetate buffer for 20 min, after which several scans were collected. After equilibration of the film in buffer, a 5 μM protein solution was injected into the measurement cell and incubated with the films in absence of flow until little change in NR was observed on a timescale of several hours. The incubation time therefore varied somewhat among the samples. The NR studies were performed at room temperature and also at the optimal temperature for each CD (65°C and 75°C for Cel9A and Cel5A, respectively). The sample cell was heated by circulating a heating fluid through copper blocks placed underneath and on top of the sample cell. The sample cell and copper blocks were enclosed in a Styrofoam box containing thin aluminum foil windows. The temperature of the sample cell was continuously monitored with a thermocouple.

## Abbreviations

CBM: Carbohydrate-binding module; CD: Catalytic domain; QCM-D: Quartz crystal microbalance with dissipation; NR: Neutron reflectometry; MCC: Microcrystalline cellulose; IL: Ionic liquid; IL-MCC: Ionic-liquid pretreated microcrystalline cellulose; RT: Room temperature; KO: knockout; SLD: Scattering length density; CMC: Carboxymethylcellulose; TMSC: Trimethylsilylcellulose; DNS: dinitrosalicylic acid; PCR: polymerase chain reaction.

## Competing interests

The authors declare that they have no competing interests.

## Authors’ contributions

VRO carried out the enzyme assays, collected NR data, helped in the design and interpretation of experiments, and drafted the manuscript. RAH and RBE helped carry out enzyme assays and participated in the design and interpretation of experiments. EYK helped to design and construct the vectors and chimeras. BCV and GC assisted with NR experiments. MSK designed and carried out the NR experiments, and helped to draft the manuscript. PDA, KLS, and MZH assisted in design, interpretation, and coordination of experiments and edited the manuscript. BAS conceived of the project, participated in the coordination, design, and interpretation of experiments, and edited the manuscript. DTE conceived of the project, assisted in the design and interpretation of experiments, and helped to draft and edit the manuscript. All authors read and approved the final manuscript.

## Supplementary Material

Additional file 1 **Enzyme characterization data, neutron reflectivity data, scattering length density profiles, and a sample volume fraction uncertainty profile can be found in Additional file****1****: Figures S1 through S15.** Click here for file
