# Supplementary material for: Addition of a carbohydrate-binding module enhances cellulase penetration into cellulose substrates
Source: Biotechnol Biofuels. 2013 Jul 3;6:93. doi: 10.1186/1754-6834-6-93 (PMC3716932; doi:10.1186/1754-6834-6-93)
Supplement: Additional file 1 — Enzyme characterization data, neutron reflectivity data, scattering length density profiles, and a sample volume fraction uncertainty profile can be found in Additional file1: Figures S1 through S15. [file 1754-6834-6-93-S1.pdf]

Additional File 1

Addition of a carbohydrate-binding module enhances cellulase penetration into cellulose substrates

Vimalier Reyes-Ortiz[1,2], Richard A Heins[1,3], Gang Cheng[1,3], Edward Y Kim[5], Briana C Vernon[1,4], Ryan B Elandt[1], Paul D Adams[1,2,6], Kenneth L Sale[1,3], Masood Z Hadi[1,3], Blake A Simmons[1,3], Michael S Kent[1,4], Danielle Tullman-Ercek[1,2,5,6,*]

[1] Deconstruction Division, Joint BioEnergy Institute, Emeryville, CA 94608, US

[2] Department of Bioengineering, University of California, Berkeley, CA 94720, US

[3] Sandia National Laboratories, Livermore, CA 94550, US

[4] Sandia National Laboratories, Albuquerque, NM 87185, US

[5] Department of Chemical and Biomolecular Engineering, University of California, Berkeley, CA 94720, US

[6] Physical Biosciences Division, Lawrence Berkeley National Laboratory, Berkeley, CA 94720, US

[*] Corresponding author. Physical Biosciences Division, Lawrence Berkeley National Laboratory, Berkeley, CA 94720, US

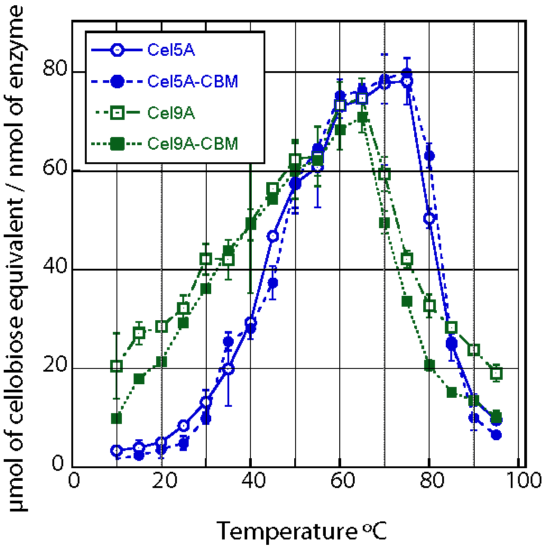

Figure S1: Addition of the CBM does not affect the optimal temperature of the wild type catalytic domain. Enzymatic activities of the wild type and chimeric cellulases on carboxymethyl cellulose (CMC) are shown as a function of temperature. Each cellulase was run at its optimal pH (4.8 and 5.5 for Cel5A and Cel9A, respectively).

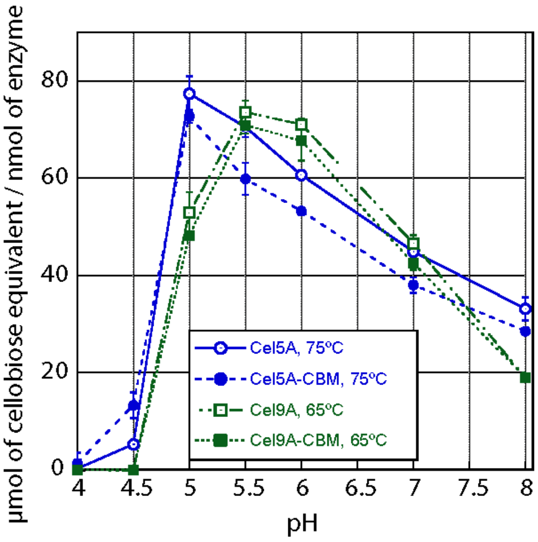

Figure S2: Addition of the CBM does not affect the behavior of the catalytic domain at various pHs. Enzymatic activities are shown for the wild type and chimeric cellulases on carboxymethyl cellulose (CMC) as a function of pH. Each cellulase was run at its optimal temperature (75°C and 65°C for Cel5A and Cel9A, respectively).

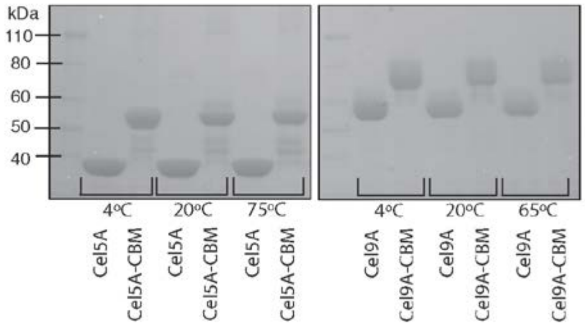

Figure S3: The linker between the catalytic domain (CD) and the carbohydrate binding domain (CBM) is stable at high temperatures. Stability of the linker was tested by splitting each of the chimeric enzyme samples into three aliquots, and then incubating all of the enzymes at their optimal temperatures (T_{opt}), 4ºC, or 20ºC for 24 hrs. All samples were then run on a polyacrylamide gel, which was subsequently incubated with Coomassie stain.

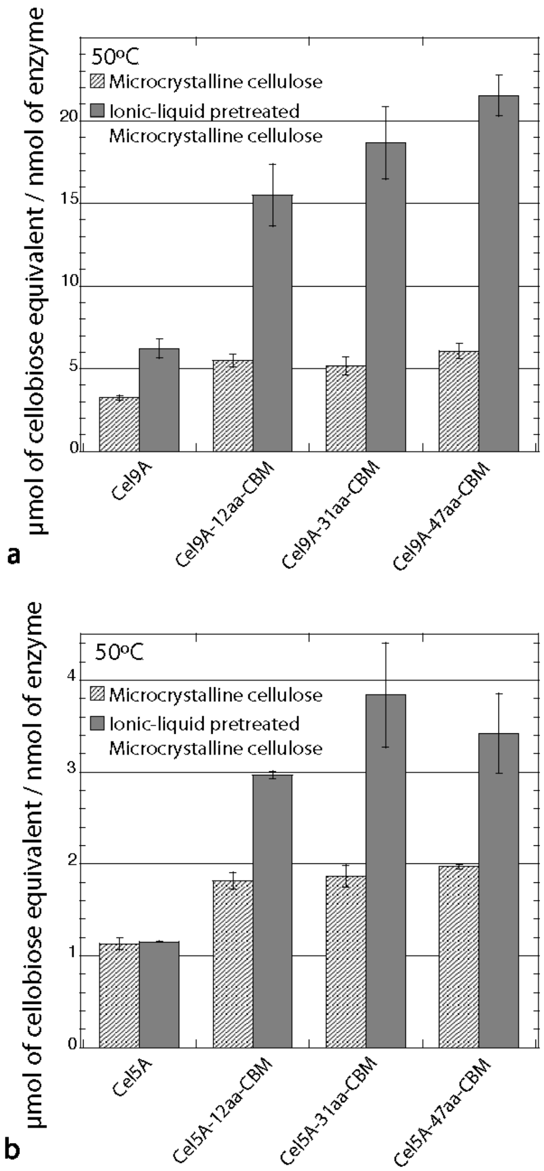

Figure S4: Chimera linker length does not substantially affect hydrolysis of cellulosic substrates. Two insoluble substrates, microcrystalline cellulose (MCC) and ionic liquid-pretreated MCC (IL-MCC), were used for this assay. a) Cel9A, Cel9A-12aa-CBM, Cel9A-31aa-CBM, and Cel9A-47aa-CBM; b) Cel5A, Cel5A-12aa-CBM, Cel5A-31aa-CBM, and Cel5A-47aa-CBM. Cellulases were loaded at 200 nM with either 50 mg/ml MCC or IL-MCC at 50°C for 24 h, and DNS assay was used to quantify reducing sugars with a cellobiose standard curve. The number of amino acids (aa) in each linker is denoted by “12aa”, “31aa” and “47aa”.

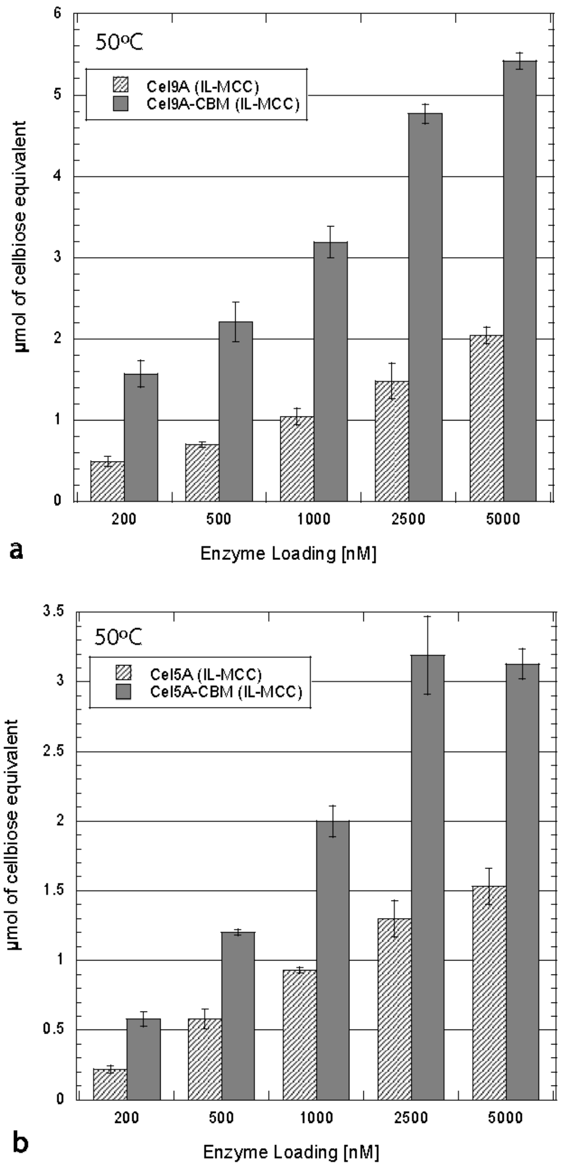

Figure S5: The fold increase in activity of chimeric cellulases (CD-CBM) compared to the wild type cellulases (CD alone) is comparable over a wide range of enzyme concentration. Ionic liquid-pretreated MCC (IL-MCC) was used for this assay. Enzyme loadings of 200 nM to 5000 nM were tested on samples with 50 mg/ml IL-MCC at 50°C for 24 h, and DNS assay was used to quantify reducing sugars with a cellobiose standard. Results are shown for Cel9A and Cel9A-CBM (a) and Cel5A and Cel5A-CBM (b).

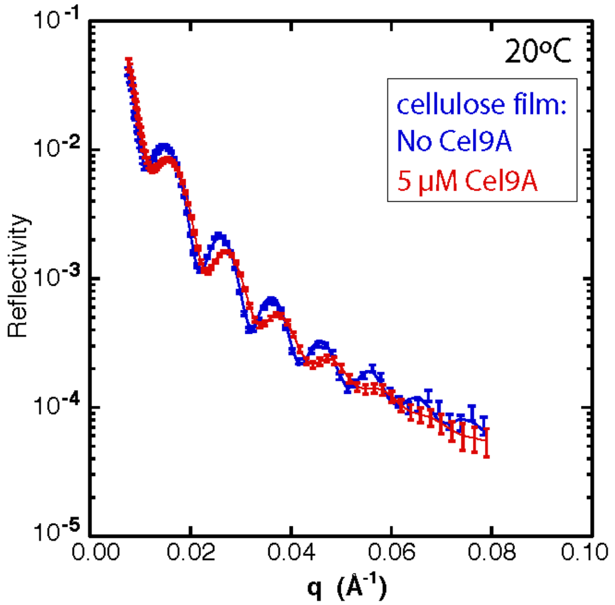

Figure S6: Neutron reflectivity data from cellulose films exposed to Cel9A at 20°C.

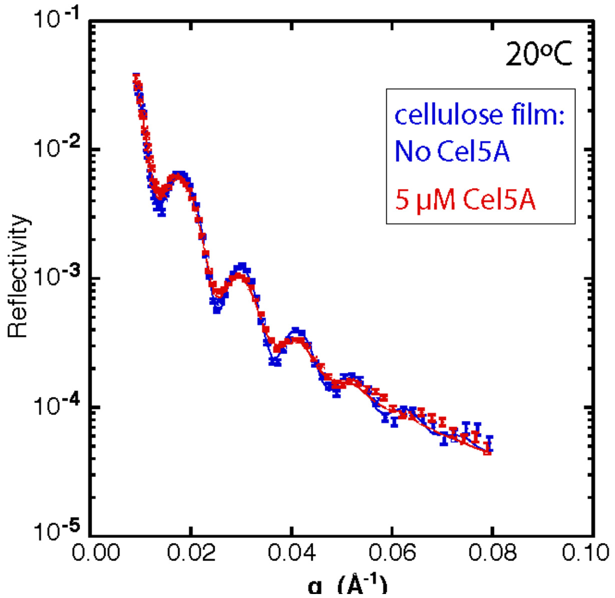

Figure S7: Neutron reflectivity data from cellulose films exposed to Cel5A at 20°C.

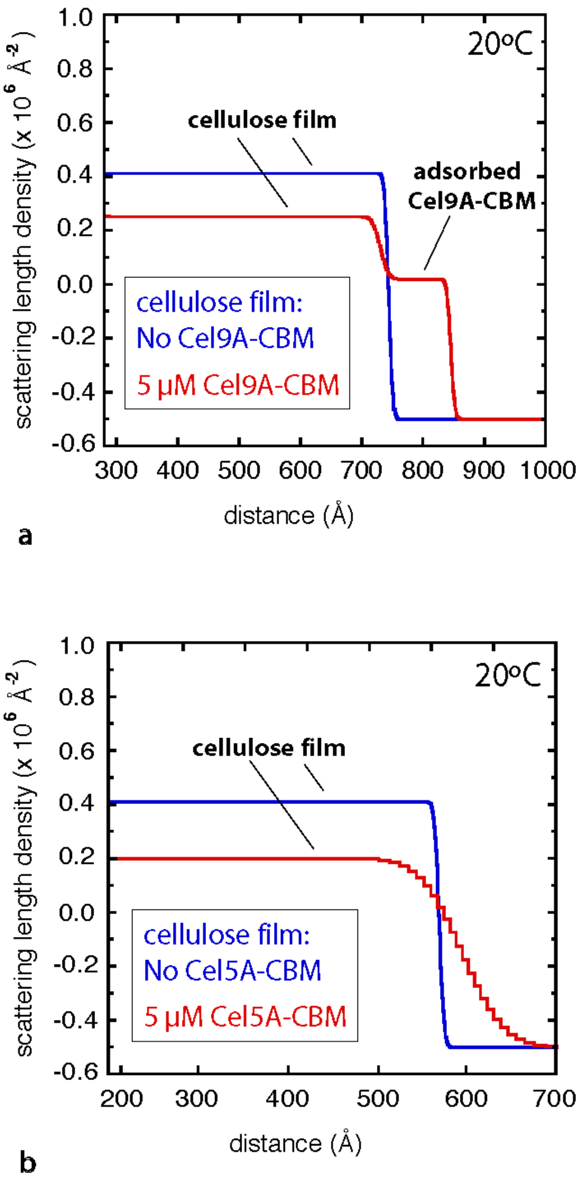

Figure S8: Scattering length density (SLD) profiles with respect to distance for Cel9A-CBM (a) and Cel5A-CBM (b) at 20°C. The SLD profile for Cel9A-CBM shows an additional layer at the film-solution interface that we attribute to adsorbed protein.

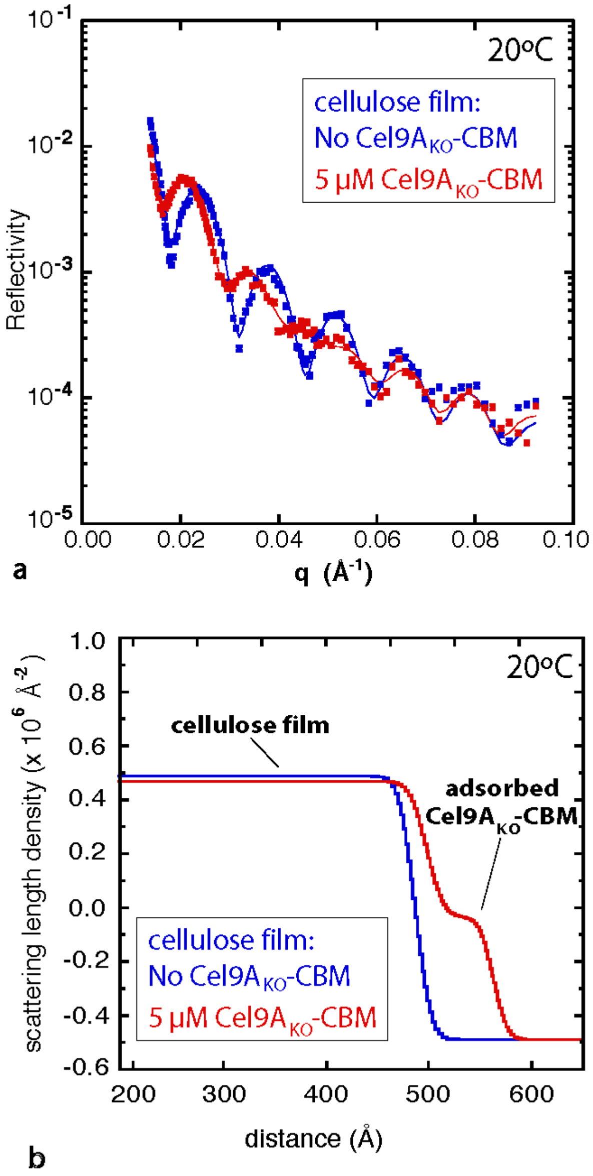

Figure S9: Neutron reflectivity data (a) and SLD profiles (b) for a cellulose film before and after incubation with 5 μM Cel9A$_{KO}$-CBM at 20°C. The SLD profile for Cel9A$_{KO}$-CBM shows an additional layer at the film-solution interface that we attribute to adsorbed protein.

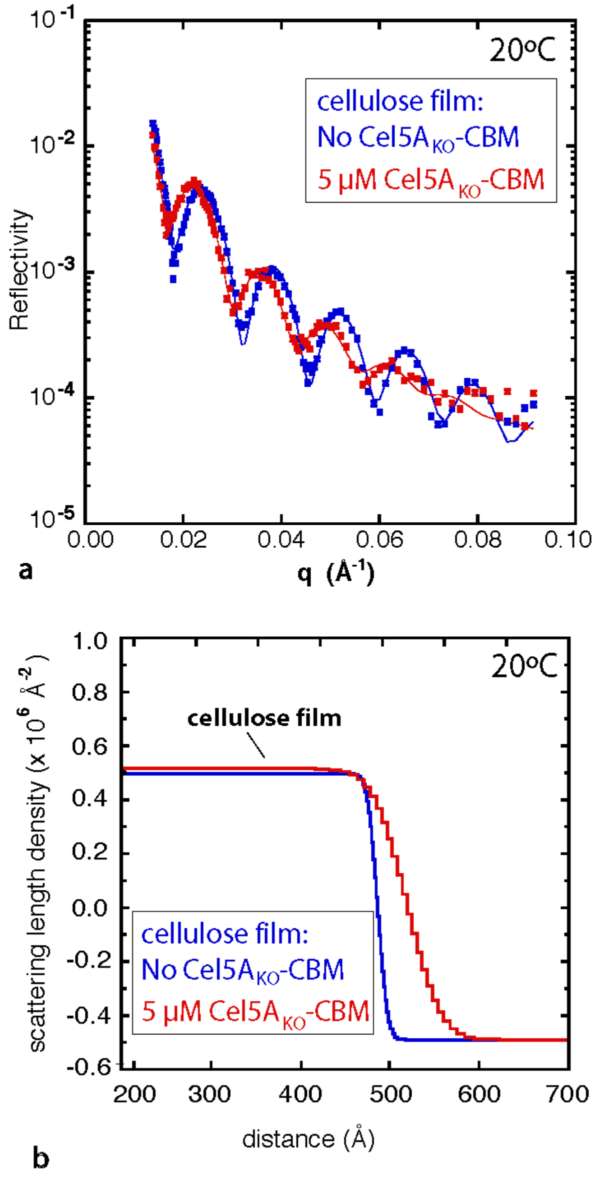

Figure S10: Neutron reflectivity data (a) and SLD profiles (b) for a cellulose film before and after incubation with 5 µM Cel5A$_{KO}$-CBM at 20°C. The cellulose films are indicated.

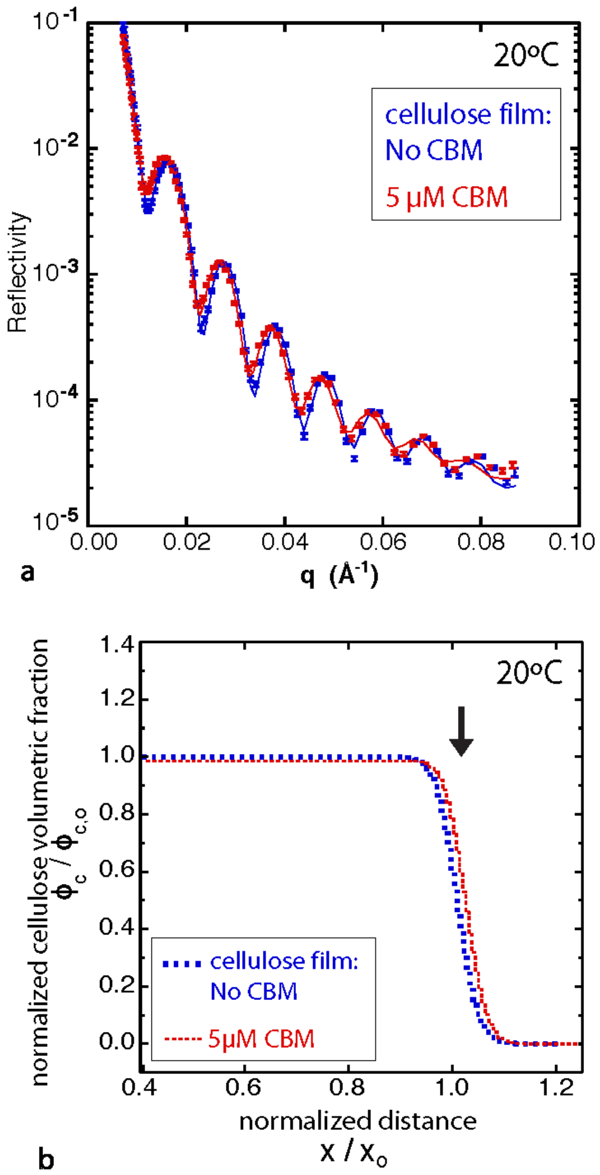

Figure S11: Neutron reflectivity data (a) and normalized cellulose volume fraction profiles (b) for a cellulose film before and after incubation with the CBM at 5 µM and 20°C.

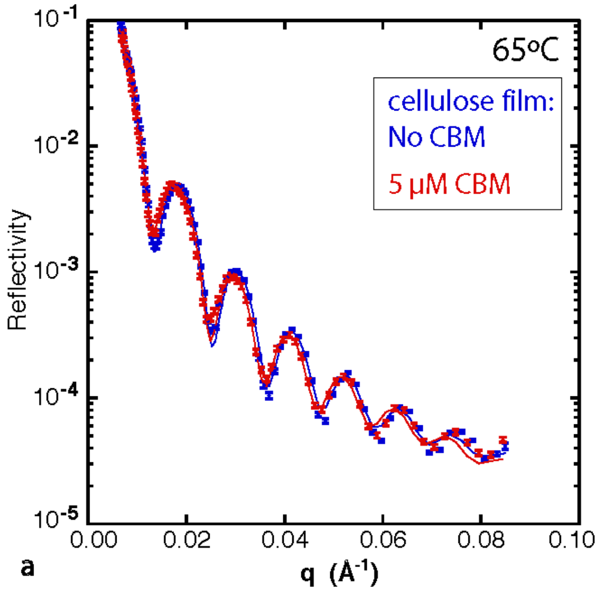

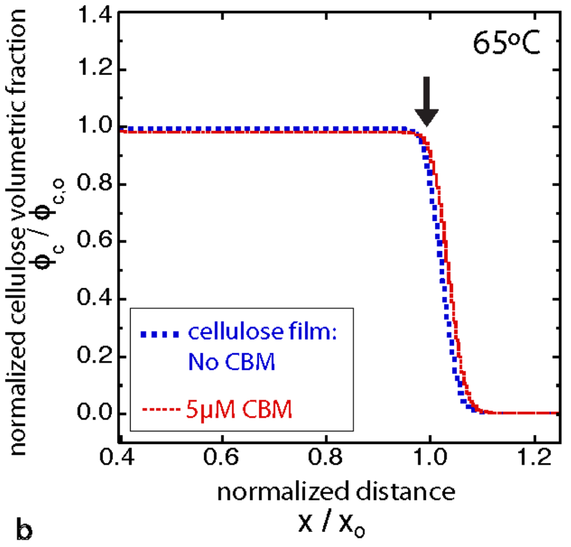

Figure S12: Neutron reflectivity data (a) and normalized cellulose volume fraction profiles (b) for a cellulose film before and after incubation with the CBM at 5 μM and 65°C.

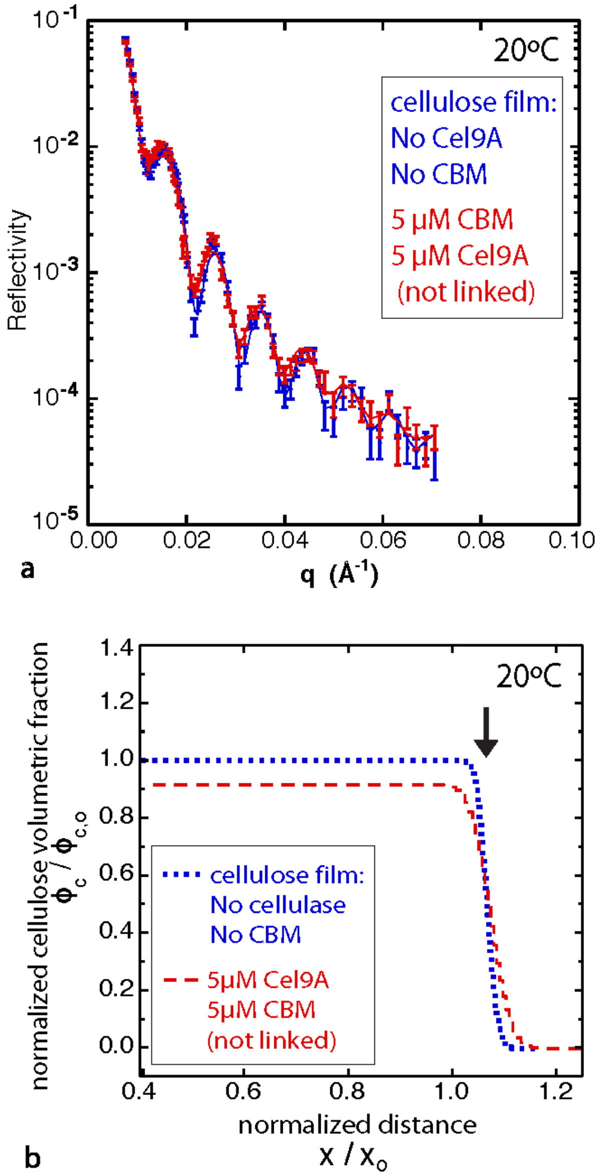

Figure S13: Neutron reflectivity data (a) and normalized cellulose volume fraction profiles (b) for a cellulose film before and after incubation with a solution containing 5 µM Cel9A and 5 µM CBM at 20°C.

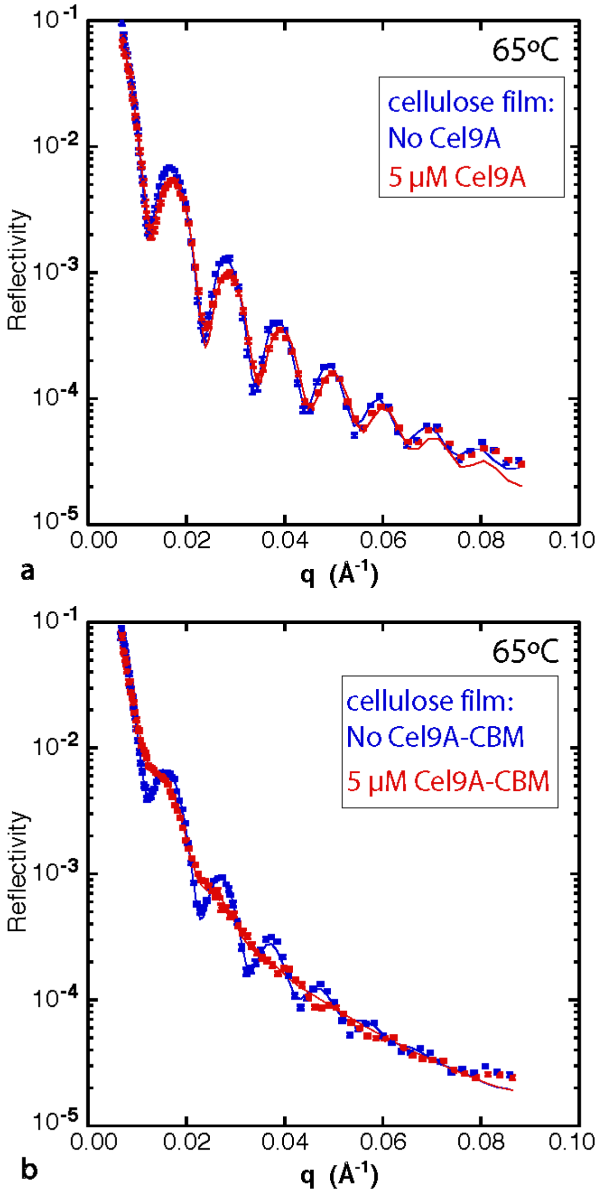

Figure S14: Neutron reflectivity data from cellulose films before and after incubation with a) 5 µM Cel9A and b) 5 µM Cel9A-CBM at 65°C.

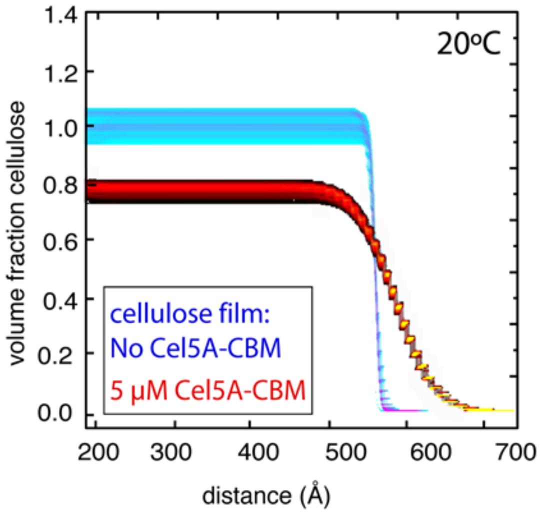

Figure S15: Plot showing volume fraction profiles with the uncertainty limits indicated as multicolored bands for Cel5A-CBM (yellow/red/black) and Cel5A (cyan/blue) at 20°C. The uncertainty bands shown in this plot are representative of the uncertainty in the SLD plots for each NR experiment performed in this study.
